# Vascular and renal function of cGMP signalling

**DOI:** 10.1186/1471-2210-11-S1-O17

**Published:** 2011-08-01

**Authors:** Jens Schlossmann, Elisabeth Schinner, Matthias Desch, Bernhard Hieke, Petra Rothammer, Katharina Salb, Andrea Schramm

**Affiliations:** 1 Lehrstuhl für Pharmakologie und Toxikologie, Universität Regensburg, Germany

## Background

Signalling by NO/cGMP is significant for regulation of cardiovascular and renal function.

## Results

For the functional analysis of cGMP signalling, transgenic murine mutants were used. cGKI signalling pathways include interaction of the cGKIβ-isozyme with the inositol 1,4,5-trisphosphate receptor I (IP_3_RI) associated protein cGMP kinase substrate (IRAG). NO/cGMP and ANP/cGMP-dependent relaxation of aortic smooth muscle was strongly affected in IRAG-deficient mice. NO/ANP-dependent inhibition of intracellular calcium release was suppressed in IRAG-deficient vascular smooth muscle cells (VSMC). Furthermore, reduction of store operated calcium entry by cGMP was affected in IRAG-KO VSMC. Basal mean arterial blood pressure, heart rate and activity were not changed in IRAG knockout mice. However, under pathophysiological conditions like sepsis (induced by E. coli lipopolysaccharide application) IRAG knockout mice were resistant to blood pressure reduction.

## Conclusion

These results suggested that cGKI/IRAG signalling is a predominant signal transduction pathway of NO/ANP/cGMP which may be involved in vascular diseases including pathophysiological modulation of blood pressure. Further results provided evidence that cGMP signalling is essential for prevention of renal diseases suppressing interstitial fibrosis.

